# Changes in Astroglial Markers in a Maternal Immune Activation Model of Schizophrenia in Wistar Rats are Dependent on Sex

**DOI:** 10.3389/fncel.2015.00489

**Published:** 2015-12-24

**Authors:** Daniela F. de Souza, Krista M. Wartchow, Paula S. Lunardi, Giovana Brolese, Lucas S. Tortorelli, Cristiane Batassini, Regina Biasibetti, Carlos-Alberto Gonçalves

**Affiliations:** Departamento de Bioquímica, Instituto de Ciências Básicas da Saúde, Universidade Federal do Rio Grande do SulPorto Alegre, Brazil

**Keywords:** animal model, astrogliosis, GFAP, lipopolysaccharide, schizophrenia, S100B

## Abstract

Data from epidemiological studies suggest that prenatal exposure to bacterial and viral infection is an important environmental risk factor for schizophrenia. The maternal immune activation (MIA) animal model is used to study how an insult directed at the maternal host can have adverse effects on the fetus, leading to behavioral and neurochemical changes later in life. We evaluated whether the administration of LPS to rat dams during late pregnancy affects astroglial markers (S100B and GFAP) of the offspring in later life. The frontal cortex and hippocampus were compared in male and female offspring on postnatal days (PND) 30 and 60. The S100B protein exhibited an age-dependent pattern of expression, being increased in the frontal cortex and hippocampus of the MIA group at PND 60, while at PND 30, male rats presented increased S100B levels only in the frontal cortex. Considering that S100B secretion is reduced by elevation of glutamate levels, we may hypothesize that this early increment in frontal cortex tissue of males is associated with elevated extracellular levels of glutamate and glutamatergic hypofunction, an alteration commonly associated with SCZ pathology. Moreover, we also found augmented GFAP in the frontal cortex of the LPS group at PND 30, but not in the hippocampus. Taken together data indicate that astroglial changes induced by MIA are dependent on sex and brain region and that these changes could reflect astroglial dysfunction. Such alterations may contribute to our understanding of the abnormal neuronal connectivity and developmental aspects of SCZ and other psychiatric disorders.

## Introduction

Schizophrenia (SCZ) is a chronic and debilitating illness that affects about 1% of the world population, with the onset of the manifestation occurring typically in late adolescence or in early adulthood (Monji et al., 2013). The incidence of SCZ is significantly higher in men than in women (male: female ratio = 1.4) (Aleman et al., 2003; McGrath et al., 2008). Nevertheless, the etiology of SCZ remains unclear, although numerous findings indicate that neurodevelopmental factors contribute to its pathophysiology (Murray et al., 1992; Knuesel et al., 2014). Among these factors are included prenatal exposure to infection agents such as viruses (Kneeland and Fatemi, 2012) and gram-negative bacteria (Babulas et al., 2006; Sørensen et al., 2009; Khandaker et al., 2012).

The MIA animal model is used to study how an insult directed at the maternal host can have adverse effects on the fetus, leading to behavioral and neurochemistry changes later in life, specifically within abnormal exploration and social behaviors, cytokine levels and gene regulation (Ashdown et al., 2006; Fatemi et al., 2008; Meyer et al., 2009). Interestingly, prenatal exposure to the viral mimetic polyinosinic-polycytidylic acid changed behavioral flexibility of offspring rats, in a sex-dependent manner (Zhang et al., 2012). Systemic administration of the bacterial endotoxin, LPS, is a widely used and accepted MIA model that emulates immune activation and subsequent release of immunoregulatory, cytotoxic and inflammatory cytokines secondary to gram-negative bacterial infections (Borrell et al., 2002). Furthermore, inflammatory signals have been described in the hippocampus and cerebral cortex in postmortem studies of SCZ patients and MIA models (Beumer et al., 2012).

Changes in glial cells seem to be closely related to the pathology of SCZ (Cotter et al., 2001; Bernstein et al., 2009; Beumer et al., 2012). Astrocytes, the most abundant glial cells, are involved, together with microglia, in brain immune activation, as well as antioxidant defenses and glutamatergic neurotransmission (Takuma et al., 2004; Liu et al., 2011). S100B, a protein mainly expressed and secreted by astrocytes in the CNS, has been proposed as a marker of brain damage (Marchi et al., 2004; Gonçalves et al., 2008; Koh and Lee, 2014) and several studies have suggested that S100B is altered in neurological and psychiatric disorders (Ashraf et al., 1999; Lara et al., 2001; Steiner et al., 2011). Corroborating the idea of the neuroinflammatory basis of SCZ, and the involvement of the S100B protein in its pathogenesis, we recently showed that cerebrospinal fluid (CSF) S100B is increased by intracerebroventricular or intraperitoneal LPS administration (Guerra et al., 2011). Furthermore we observed that S100B secretion stimulated by cytokines *in vitro* is prevented by antipsychotics (de Souza et al., 2013).

Brain inflammation involving astrogliosis (characterized by over expression of GFAP and/or astrocyte hypertrophy) has been observed in the offspring of models of MIA, in IL-6 or LPS-treated mothers (Samuelsson et al., 2006; Hao et al., 2010). However, the role of GFAP in SCZ is controversial; some studies have found no changes or decreased GFAP content in the cortex and cerebellum of schizophrenic patients (Falkai et al., 1999; Rajkowska et al., 2002). On the other hand, there is evidence that this protein might be significantly augmented in demented schizophrenic patients, when compared to non-demented patients (Arnold et al., 1996).

Additionally, studies suggest that the altered regulation of fundamental mechanisms of anti-oxidant defense, where astrocytes are key elements (Takuma et al., 2004), may contribute to the pathogenesis of SCZ and related disorders (Floyd, 1999; Chauhan and Chauhan, 2006; Boskovic et al., 2011). In fact, analyses of the molecular mechanisms underlying oxidative stress suggest that cognitive dysfunction may be associated with an imbalance in the generation and clearance of ROS (Bitanihirwe and Woo, 2011) and MIA models support the role of oxidative/nitrosative stress in SCZ (Venkatasubramanian and Debnath, 2013)

In this study, we evaluated whether the administration of LPS in rat dams during late pregnancy affects the main astroglial markers, S100B and GFAP, in the frontal cerebral cortex and hippocampus of the dams’ offspring during later life. S100B levels were also investigated in CSF and serum. We compared the offspring at 30 and 60 days to evaluate the possible differences between juvenile and adult rats and also investigated the existence of differences between male and female offspring. We also investigated the oxidative/nitrosative stress parameters, NO and GSH contents, in this model.

## Materials and Methods

### Animals

Female Wistar rats from our breeding colony (Department of Biochemistry, UFRGS, Porto Alegre, Brazil), weighing 216–263 g each, were used, and maintained under controlled light and environmental conditions (12 h light/12 h dark cycle at a constant temperature of 22 ± 1°C), with free access to commercial chow and water. The fertility cycle of the rats was controlled, and, when on proestrus, they were mated overnight. In the morning, vaginal secretion was collected for analysis. If spermatozoa were found in the morning, the day was designated as the first day of pregnancy. All animal experiments were carried out in accordance with the National Institute of Health Guide for the Care and Use of Laboratory Animals (NIH Publications No. 80-23) revised in 1996 and followed the regulations of the local animal housing authorities.

The study has been approved by the Comite de Etica no Uso de Animais (CEUA), UFRGS, number 18672.

### LPS Administration to Pregnant Rats

For gestational LPS treatment, timed pregnant Wistar rats were injected on days 18 and 19 of pregnancy, as follows: six pregnant rats were injected intraperitoneally with 500 μg/kg LPS (from *Escherichia coli*, serotype 055:B5, Sigma) and five were injected with a corresponding volume of sterile saline (control), once daily. Females were kept separate and with free access to their own litters. Rats from both groups (control and LPS) were born healthy and the numbers of offspring were normal. The offspring rats were weaned at 21 days old and were housed separately according to sex. The experiments were performed using male and female rats from each litter. Rats had free access to food and water. All the experiments were performed between 12:00 h and 17:00 h. In order to analyze the differences between young and adult rats, experiments were performed at PND 30 and PND 60 (Cai et al., 2000).

### Obtaining CSF, Serum and Hippocampal Samples

Animals were anesthetized with ketamine/xylazine (75 and 10 mg/kg, respectively, i.p.) and then positioned in a stereotaxic holder; CSF was obtained by cisterna magna puncture using an insulin syringe (27 gage × 1/2 inch length). The blood samples were collected by careful intracardiac puncture, using a 5-mL non-heparinized syringe to obtain 3 mL of blood. Blood samples were incubated at room temperature (25°C) for 5 min and centrifuged at 3200 rpm for 5 min to obtain serum. CSF and serum were frozen (-20°C) until further analysis, at most for 2 weeks. The animals were killed by decapitation, and the brains were removed and placed in cold saline medium with the following composition (in mM): 120 NaCl; 2 KCl; 1 CaCl2; 1 MgSO4; 25 HEPES; 1 KH2PO4 and 10 glucose, adjusted to pH 7.4. The hippocampi and frontal cortex were dissected and transverse slices of 0.3 mm were obtained using a McIlwain Tissue Chopper. Slices were then frozen at -20°C (for measurement of GFAP and S100B) or -80°C (for measurement GSH and NO), at most for 2 weeks.

### ELISA for S100B

The S100B concentration was determined in the hippocampal and cortical samples, in addition to serum and CSF from offspring at PND 30 and PND 60. S100B levels were determined by ELISA, as described previously (Leite et al., 2008). Briefly, 50 μL of sample plus 50 μL of Tris buffer were incubated for 2 h on a microtiter plate, previously coated with anti-S100B monoclonal antibody (SH-B1, from Sigma). Anti-S100 polyclonal antibody (from DAKO) was incubated for 30 min and then peroxidase-conjugated anti-rabbit antibody was added for a further 30 min. The color reaction with *o*-phenylenediamine was measured at 492 nm. The standard S100B curve ranged from 0.002 to 1 ng/mL.

### ELISA for GFAP

Enzyme-linked immunosorbent assay for GFAP was carried out by coating the microtiter plate with 100 μL samples containing 20 ng of protein for 24 h at 4°C. Incubation with a polyclonal anti-GFAP from rabbit (GE Healthcare) for 1 h was followed by incubation with a secondary antibody conjugated with peroxidase for 1 h, at room temperature. A colorimetric reaction with *o*-phenylenediamine was measured at 492 nm. The standard human GFAP (from Calbiochem) curve ranged from 0.1 to 5 ng/mL.

### Immunohistochemistry for GFAP and NeuN

Rats were anesthetized using ketamine/xylazine and were perfused through the left cardiac ventricle with 200 mL of saline solution, followed by 200 mL of 4% paraformaldehyde in 0.1 M phosphate buffer, pH 7.4. The brains were removed and left for post-fixation in the same fixative solution at 4°C for 24 h. Subsequently, the material was cryoprotected by immersing the brain in 30% sucrose in phosphate buffer at 4°C. The brains were sectioned (50 μm) on a cryostat (Leitz). The sections were then preincubated in 2% bovine serum albumin (BSA) in phosphate-buffered saline (PBS) containing 0.4% Triton X-100 for 30 min and incubated with polyclonal anti-GFAP from rabbit or -NeuN from mouse, diluted 1:500 in 0.4% BSA in PBS-Triton X-100, for 48 h at 40°C. After washing several times, tissue sections were incubated with a secondary antibody Alexa Fluor 488 (goat anti-rabbit-IgG; green fluorescence) and Alexa Fluor 568 (goat anti-mouse-IgG; red fluorescence) diluted 1:500 in PBS, at room temperature for 2 h. Afterward, the sections were mounted on slides with Fluor Save and covered with coverslips. Samples were quantified according to (Centenaro et al., 2011). Briefly, images were viewed with an Olympus microscope with a digital camera and then transferred to a computer. Then, the GFAP and NeuN immunoreactivity was evaluated by means of regional semi-quantitative optical densitometry, using Image J Software 1.42q (Wayne Rasband, National Institutes of Health, USA). Five images were analyzed in the stratum radiatum of the CA1 in the hippocampus from each animal (four fields were analyzed per section).

### Glutathione Content Assay

Glutathione levels (nmol/mg protein) were measured, as described previously (Anderson, 1985). Slices were homogenized and assayed in 10 volumes of 100 mM sodium phosphate buffer, pH 8.0, containing 5 mM EDTA and protein was precipitated with 1.7% meta-phosphoric acid. Supernatant was assayed with *o*-phthaldialdeyde (1 mg/mL methanol) at room temperature for 15 min. Fluorescence was measured using excitation and emission wavelengths of 350 and 420 nm, respectively. A calibration curve was performed with standard GSH solutions (0–500 μM).

### Nitric Oxide (NO) Production

Nitric Oxide metabolites, NO^3-^ (nitrate) and NO^2-^ (nitrite) were determined according to (Hu et al., 1996). Briefly, homogenates from one hippocampus were mixed with 25% trichloroacetic and centrifuged at 1,800 × *g* for 10 min. The supernatant was immediately neutralized with 2 M potassium bicarbonate. NO^3-^ was reduced to NO^2-^ by nitrate reductase. The total NO^2-^ in the supernatant was measured by a colorimetric assay at 540 nm, based on the Griess reaction. A standard curve was performed using sodium nitrate (0–50 μM).

### Protein Determination

Protein content was measured by Lowry’s method using BSA as standard (Peterson, 1977).

### Statistical Analysis

Parametric data are reported as means ± standard error and were analyzed by two-way ANOVA (followed by Bonferroni’s test). Values of *p* < 0.05 were considered to be significant.

## Results

### Prenatal LPS Treatment Increases S100B in the Frontal Cortex and Hippocampus of Offspring Rats

**FIGURE 1:**
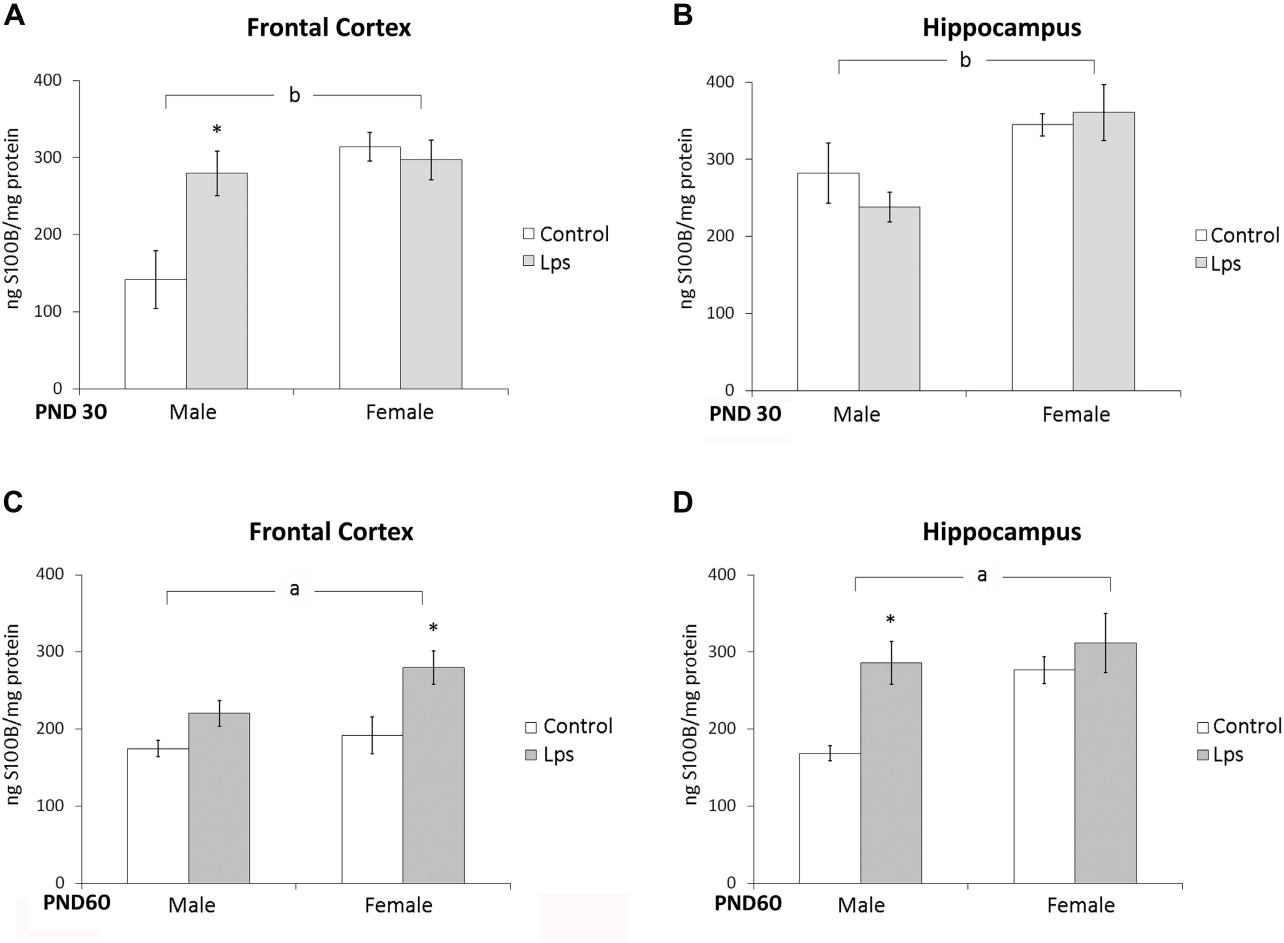
**Prenatal LPS treatment increases S100B in the frontal cortex and hippocampus of offspring rats.** Cortical or hippocampal slices from PND 30 (**A,B**, respectively) and PND 60 Wistar rats prenatally exposed to LPS (**C,D**, respectively); S100B was measured by ELISA. Data are expressed as means ± standard error (LPS group, *N* = 10; control group, *N* = 10), the measurements were performed in triplicate. ^a^Significant effect of prenatal treatment and ^b^significant effect of sex (Two-way ANOVA *p* < 0.05). ^∗^Significantly different from respective control (Bonferroni’s *post hoc*, *p* < 0.05).

S100B content was measured in the frontal cortex and hippocampus of juvenile (PND 30) and adult (PND 60) rats born from mothers exposed to LPS during pregnancy. Prenatal LPS significantly changed only S100B levels in the frontal cortex of male juvenile rats (*p* < 0.05). However, a significant effect of sex on S100B levels in the frontal cortex [*F*(1,44) = 13,18; *p* = 0.0007] and in the hippocampus [*F*(1,45) = 11,89; *p* = 0.001] in juveniles rats was observed (**Figures 1A,B**).

In adult rats, a significant effect of prenatal LPS treatment on S100B immunocontent was observed in the frontal cortex [*F*(1,25) = 9,77; *p* = 0.005] and hippocampus [*F*(1,24) = 4,58; *p* = 0.04], but no effect of sex was observed [*F*(1,25) = 3,23; *p* = 0.08] and [*F*(1,24) = 3,57; *p* = 0.07] in these either of these regions (**Figures 1C,D**).

### Prenatal LPS Treatment Decreased S100B Levels in the CSF of Young Offspring Females

**FIGURE 2:**
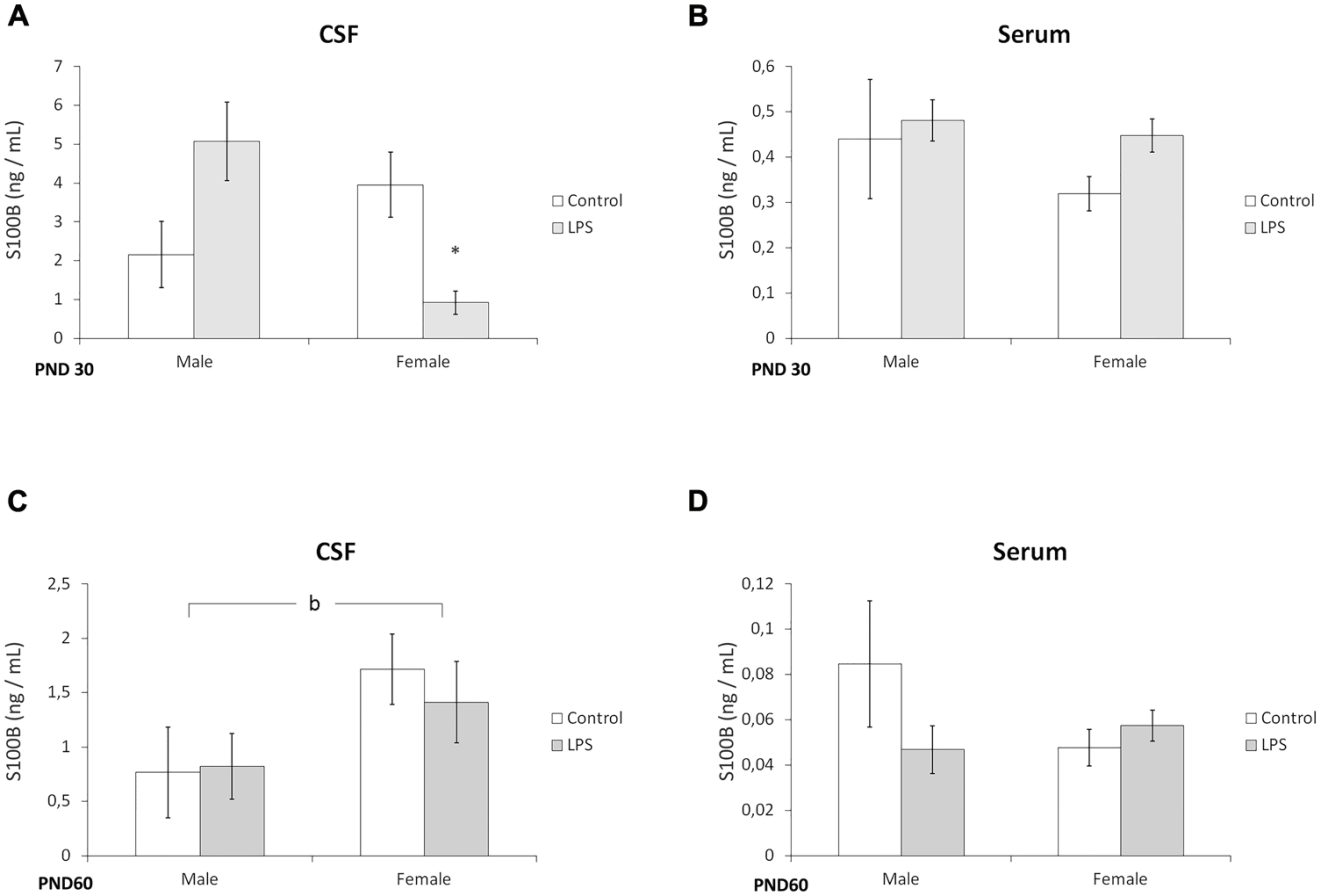
**Prenatal LPS treatment decreases S100B levels in the CSF of juvenile offspring females.** CSF and serum from PND 30 (**A,B**, respectively) and PND 60 Wistar rats prenatally exposed to LPS (**C,D**, respectively); S100B was measured by ELISA. Data are expressed as means ± standard error (LPS group, *N* = 10; control group, *N* = 10), the measurements were performed in triplicate. ^b^Significant effect of sex (*p* < 0.05) and significant interaction prenatal treatment × sex (Two-way ANOVA, *p* < 0.05). **(A)** ^∗^Significantly different from control (Two-way ANOVA followed by Bonferroni’s *post hoc*, *p* < 0.05).

In juvenile rats, a significant interaction of (LPS treatment × sex) was observed for the CSF levels of S100B [*F*(1,22) = 4,84; *p* = 0.003]. *Post hoc* analysis revealed that S100B levels were significantly lower in the females of the LPS group (*p* < 0.05), when compared with the control group (**Figure 2A**). No significant alterations were observed in the serum of juvenile offspring (**Figure 2B**).

Prenatal LPS exposure did not significantly alter S100B levels in the CSF or serum of adult offspring, however, an effect of sex on the amount of S100B in the CSF was found [*F*(1,23) = 5,96; *p* = 0.03] (**Figures 2C,D**).

### GFAP Content is Altered in Offspring Born to LPS-Treated Dams

**FIGURE 3:**
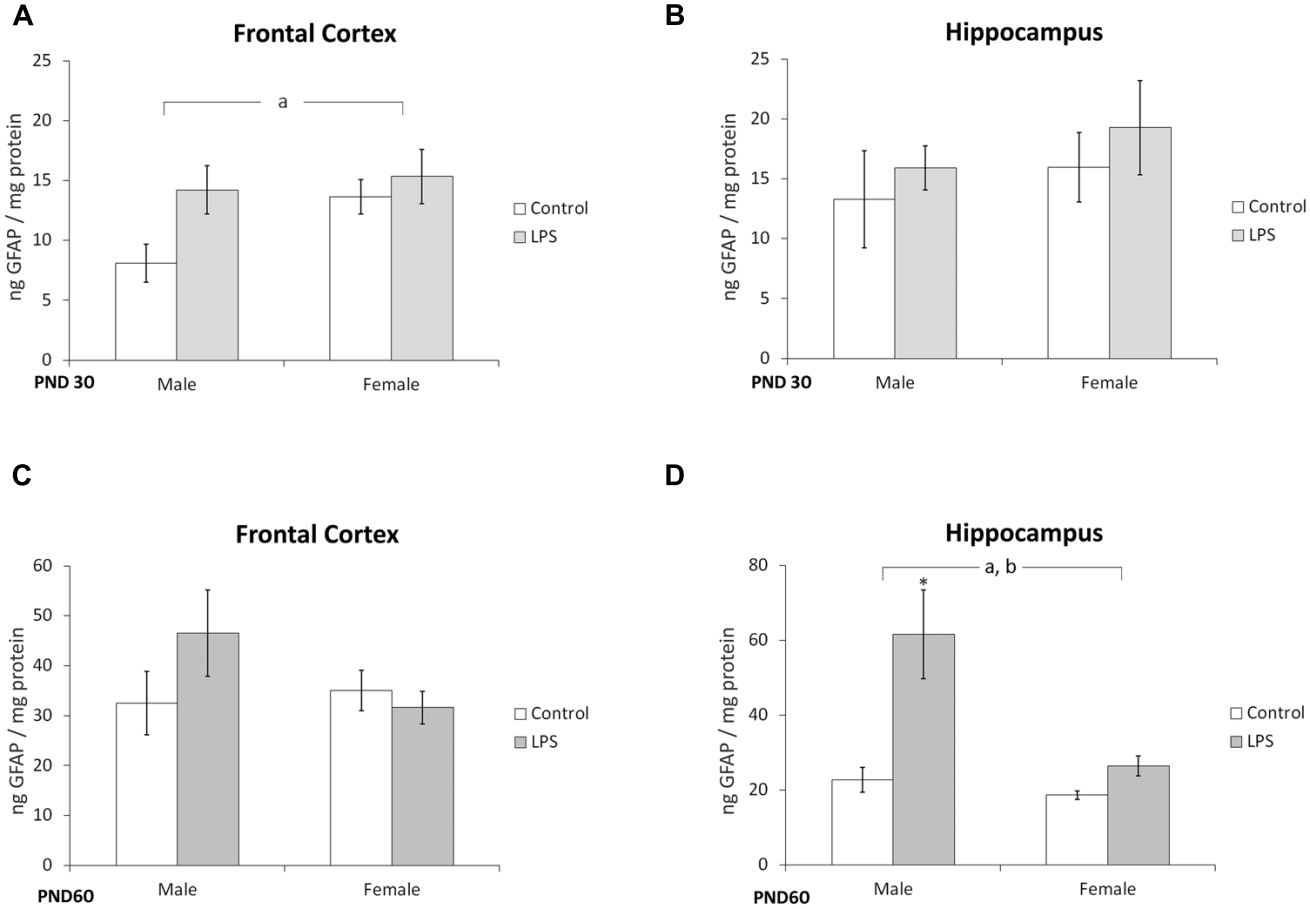
**Glial fibrillary acidic protein content is altered in offspring born to LPS-treated dams.** Cortical or hippocampal slices from PND 30 (**A,B**, respectively) and PND 60 Wistar rats, prenatally exposed to LPS (**C,D**, respectively); GFAP was measured by ELISA. Data are expressed as means ± standard error (LPS group, *N* = 10; control group, *N* = 10); the measurements were performed in triplicate. ^a^Significant effect of prenatal treatment and ^b^significant effect of sex (Two-way ANOVA, *p* < 0.05). ^∗^Significantly different from control (Bonferroni’s *post hoc*, *p* < 0.05).

A significant effect of prenatal LPS treatment [*F*(1,36) = 4,41; *p* = 0.04] on GFAP immunocontent was observed in the frontal cortex of juvenile offspring rats, but no influence of sex was observed [*F*(1,36) = 0,82; *p* = 0.8] (**Figure 3A**). No effects of LPS treatment [*F*(1,37) = 1,08; *p* = 0.30] or sex [*F*(1,37) = 0,68; *p* = 0.41] were observed in the hippocampus of juvenile rats (**Figure 3B**).

No changes were found in the frontal cortex of adult rats (**Figure 3C**). However, significant effects of LPS treatment [*F*(1,21) = 8,3; *p* = 0.008] and sex [*F*(1,21) = 5,94; *p* = 0.02] were observed in the hippocampus of adult rats (**Figure 3D**).

### Immunohistochemistry for GFAP and NeuN in the CA1 Hippocampus of LPS-Offspring Rats

**FIGURE 4:**
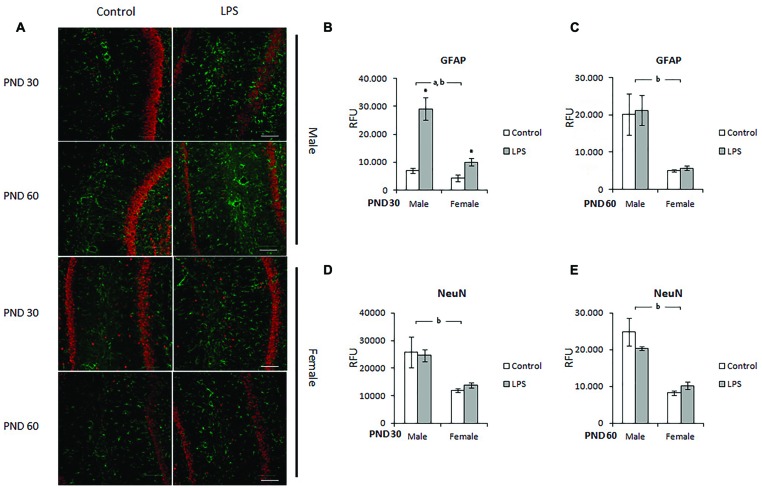
**Immunohistochemistry for GFAP and NeuN in the CA1 hippocampus of LPS-offspring rats. (A)** Shows immunohistochemistry for GFAP (green) and NeuN (red) in hipocampal slices of 30- and 60-day old Wistar rats prenatally exposed to LPS (males and females). GFAP and NeuN were then quantified in the hippocampal slices of PND 30 (**B,D**, respectively) and PND 60 Wistar rats prenatally exposed to LPS (**C,E**, respectively). Data are expressed as means ± standard error (LPS group, *N* = 5; control group, *N* = 5); the experiments were performed in triplicate. ^a^Significant effect of prenatal treatment; ^b^significant effect of sex (Two-way ANOVA, *p* < 0.05). ^∗^Significantly different from control (Bonferroni’s *post hoc*, *p* < 0.05). Scale bar = 10 mm.

In order to confirm the alterations in GFAP in the hippocampus (measured by ELISA), we localized this protein in the CA1 region using immunohistochemistry and also stained for the NeuN protein in the neuronal population. Male and female rats born to LPS-exposed dams were analyzed on PND 30 and 60 (**Figure 4A**).

After quantification, significant effects of sex [*F*(1,10) = 18,66; *p* = 0.002] and LPS treatment [*F*(1,10) = 30,62; *p* = 0.0002] were observed on the GFAP immunocontent of the CA1 hippocampus in the offspring of PND 30 rats. There was also an interaction effect between treatment and sex [*F*(1,10) = 10,51; *p* = 0.008] (**Figure 4B**). On PND 60, two-way ANOVA showed a significant effect of sex (*p* = 0.0007), but there was no effect of LPS treatment (*p* = 0.23 and 0.33, respectively) (**Figure 4C**).

Prenatal LPS did not significantly change NeuN levels in the hippocampus of juvenile or adult rats (**Figures 4D,E** respectively); however, two-way ANOVA demonstrated a significant effect of sex in both juvenile [*F*(1,12) = 33,27; *p* < 0.0001] and adult [*F*(1,14) = 45,36; *p* < 0.0001] rats.

### Offspring from LPS-Treated Dams Demonstrate Alterations in Oxidative/Nitrosative Stress

**FIGURE 5:**
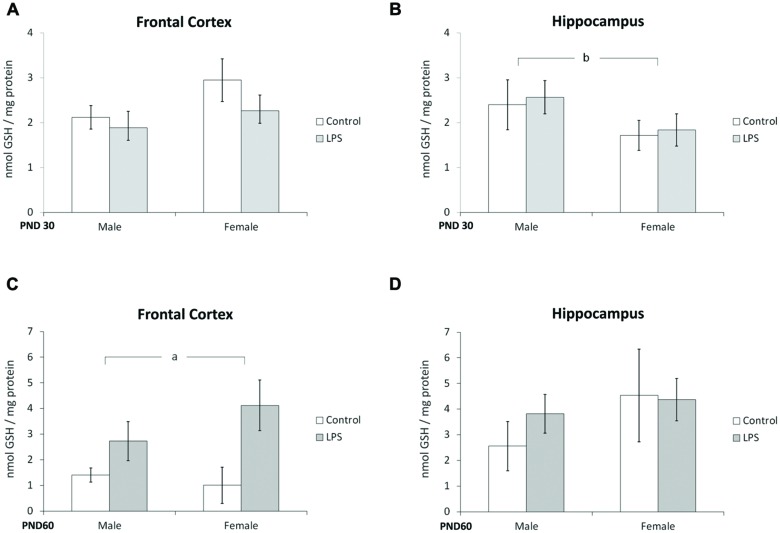
**Glutathione content is dependent on sex and modulated by prenatal treatment with LPS.** Cortical or hippocampal slices from PND 30 (**A,B**, respectively) and PND 60 Wistar rats prenatally exposed to LPS (**C,D**, respectively). Data are expressed as means ± standard error (LPS group, *N* = 10; control group, *N* = 10), the experiments were performed in triplicate. Significant interaction prenatal treatment × sex **(A)**; ^a^significant effect of prenatal treatment (Two-way ANOVA, *p* < 0.05). ^∗^Significantly different from control (Bonferroni’s *post hoc*, *p* < 0.05).

Glutathione content and NO production were used as parameters to evaluate possible oxidative stress caused by LPS prenatal exposure. Two-way ANOVA (treatment × sex) indicated no significant effect of LPS treatment or sex in the frontal cortex at PND 30 (**Figure 5A**). However, a significant effect of sex [*F*(1,40) = 4,54; *p* = 0.03] was seen in the hippocampus at PND 30 (**Figure 5B**).

A significant effect of LPS treatment on GSH content was observed in the frontal cortex at PND 60 [*F*(1,21) = 4,79; *p* = 0.04] (**Figure 5C**); no effects were found in the hippocampus at this age (**Figure 5D**).

**FIGURE 6:**
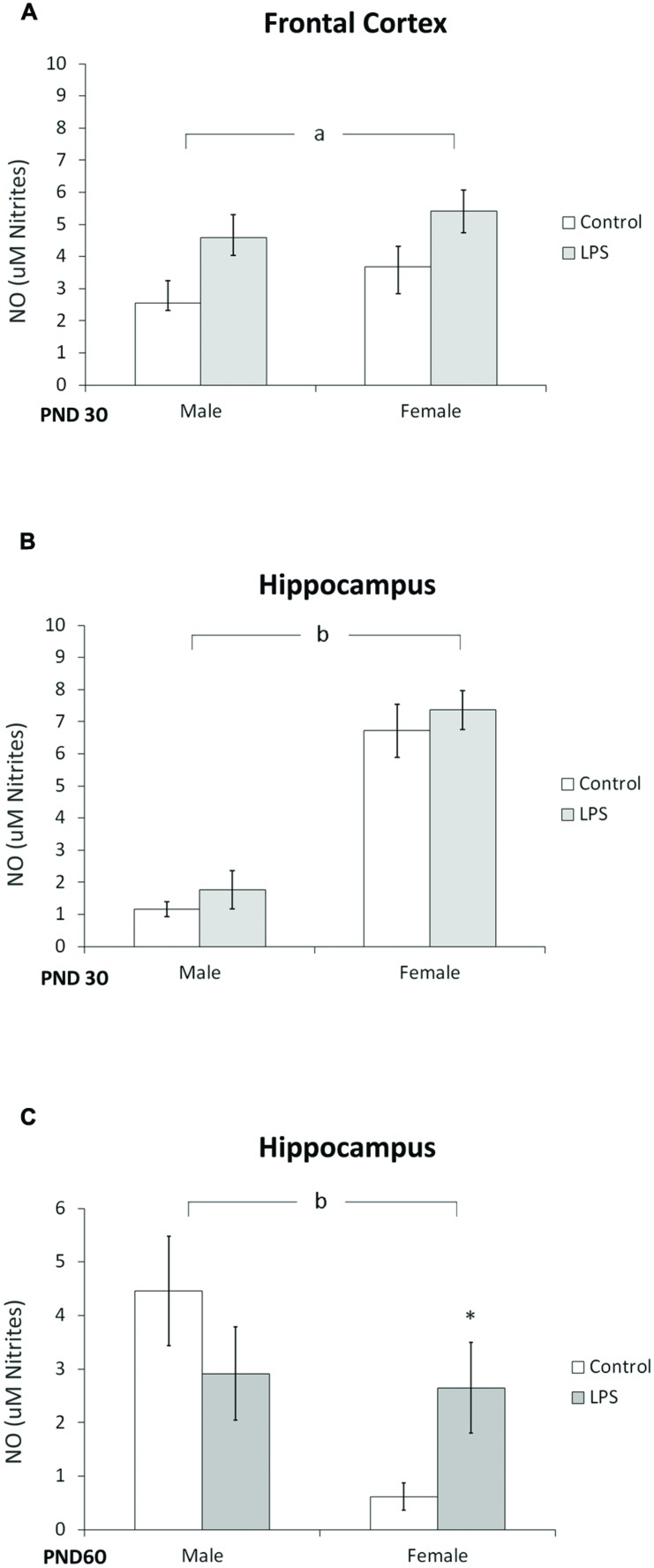
**Nitric oxide content is dependent on sex.** Cortical **(A)** and hippocampal slices from PND 30 **(B)** and hippocampal slices from PND 60 Wistar rats prenatally exposed to LPS **(C)**. Data are expressed as means ± standard error (LPS group, *N* = 10; control group, *N* = 10); experiments were performed in triplicate. ^b^Significant effect of sex (Two-way ANOVA, *p* < 0.05). ^∗^Significantly different from control (Bonferroni’s *post hoc*, *p* < 0.05).

The effect of LPS prenatal treatment on the content of NO was observed in the frontal cortex of offspring rats at PND 30 [*F*(1,14) = 7,8; *p* < 0.014] (**Figure 6A**). Furthermore, a significant effect of sex was observed in the hippocampus of juvenile [*F*(1,12) = 33,27; *p* < 0.0001] (**Figure 6B**) and adult rats [*F*(1,20) = 4,64; *p* = 0.043] (**Figure 6C**), where *post hoc* analysis indicated an increase in NO in adult females.

## Discussion

Schizophrenia is believed to involve neurochemical, metabolic activities and connectivity impairment between several brain regions, such as the prefrontal cortex and hippocampus (Cui et al., 2009; Ledoux et al., 2014). Increasing evidence suggests that an imbalance of neurodegenerative and neuroprotective factors may play a key role in this brain disorder. Of the factors that may modulate the subtle balance between cell death and survival, a role for cytokines has been consistently reported in SCZ (Mansur et al., 2012). Previous studies indicate that astroglial dysfunction could be an important element in SCZ pathology, as indicated by alterations in the markers GFAP and S100B (Fekkes et al., 2009).

S100B is a calcium-binding protein secreted by astrocytes into the synapse, where it is thought to participate in synaptic plasticity (Donato et al., 2009) and glutamatergic neurotransmission (Tramontina et al., 2006). The expression and secretion of this protein is modulated by cytokines, suggesting its involvement in the neuroinflammatory response (Schmitt et al., 2007; de Souza et al., 2009). In addition, we observed that cytokine-stimulated S100B secretion in astroglial cultures and hippocampal slices can be prevented by antipsychotics (de Souza et al., 2013). Herein, we showed that S100B has an age-dependent pattern of expression, and that it is increased in the frontal cortex and hippocampus of the LPS group at PND 60, when compared to control rats, while juvenile male rats present an increase in S100B levels only in the frontal cortex. It is important to mention that, at this age, behavioral alterations such as disruption of prepulse inhibition and deficient social interaction are associated with the SCZ model induced by MIA (Smith et al., 2007; de Souza, unpublished results).

As such, based on tissue S100B changes in the MIA model, we may suggest that a higher astroglial sensitivity/reactivity occurs in the frontal cortex of male offspring in response to prenatal LPS exposure. Accordingly, we found an increase in GFAP in the frontal cortex of the LPS group at PND 30, but not in the hippocampus. Considering that S100B secretion is reduced by elevation of glutamate levels in astrocyte cultures and brain slices (Goncalves et al., 2002; Büyükuysal, 2005; Nardin et al., 2009), we may speculate that this early increment in frontal cortex tissue of offspring males is associated with elevated extracellular levels of glutamate and glutamatergic hypofunction, commonly thought to be involved in SCZ pathology (Javitt, 2010). In support, an increment of glutamate levels has been reported in prefrontal cortex of male offspring in other MIA models induced by LPS (Connors et al., 2014) or polyinosinic:polycytidylic acid (Roenker et al., 2012). Furthermore, we found a decrease in CSF levels of S100B in females at PND 30 in the LPS group

These data are particularly interesting given the fact that the first signs of SCZ generally occur at the beginning of adulthood (Monji et al., 2009) and that an elevation of S100B has been observed in patients during the first onset of SCZ (Steiner et al., 2006). Elevations of serum S100B also have been described in SCZ patients (Wiesmann et al., 1999; Lara et al., 2001; Rothermundt et al., 2001), but no significant changes were observed in serum S100B levels at the ages analyzed in this MIA model. Furthermore, early CSF changes were not accompanied by serum changes. It is important to emphasize that changes in CSF S100B are not necessarily followed by changes in serum S100B (Gonçalves et al., 2008; Guerra et al., 2011), even though serum S100B, in SCZ, may be potentially modulated by peripheral S100B sources (Gonçalves et al., 2010; Steiner et al., 2010).

However, while increased S100B levels in patients with SCZ have been interpreted as a marker of structural damage or, alternatively, as a sign of astroglial dysfunction (Wiesmann et al., 1999; Rothermundt et al., 2001), the role of GFAP (a classical marker of astrogliosis) in psychiatric disease remains controversial (Samuelsson et al., 2006; Hao et al., 2010). We found a transitory increase of GFAP in the frontal cortex (but not in the hippocampus) at PND 30 in offspring that had been prenatally exposed to LPS, independent of sex. This hippocampal increment of GFAP was observed only at PND 60, and the effect was dependent on sex. Notably, when we looked specifically at hippocampus CA1 using immunohistochemical staining for GFAP, we found similar results at PND 60 when measuring GFAP by ELISA. Conversely, in this hippocampal region, we also observed astrogliosis at PND 30. Therefore, in spite of methodological differences, taken together these results indicate that astrogliosis in this LPS-induced MIA model is dependent on sex, time and brain region.

In parallel to immunohistochemical studies for GFAP in the CA1 hippocampus, we also stained for NeuN. We found an ontogenetic difference in this protein’s levels in males and females; however, no changes were observed at PND 30 or 60 after prenatal LPS exposure. In fact, no significant changes in the number of neurons have been described in SZC patients (Heckers and Konradi, 2002) or in MIA models of SCZ (Wolff and Bilkey, 2015). It is probable that SCZ does not occur as a consequence of a reduction in neuron number, but due to alterations in the interconnectivity between them (Selemon et al., 1995). Our data reinforce the idea that alterations in astrocytes, glial cells intimately related to synaptic plasticity, could contribute to the abnormal connectivity of neurons.

In addition to glutamatergic communication, oxidative/nitrosative stress has been associated with SCZ pathology (Dadheech et al., 2008; Do et al., 2009; Venkatasubramanian and Debnath, 2013). Astrocytes are a heterogeneous group of cells involved in the production and recycling of GSH (the main antioxidant molecule in brain tissue) (Takuma et al., 2004), and also contribute to the production of NO, which can cause nitrosative stress under certain circumstances. However, nitrosative stress in the LPS-induced MIA model has not been well characterized. We observed an increase in NO in the hippocampus of female (not males) at PND 60 following prenatal exposure to LPS. Unfortunately, we were unable to measure NO metabolites in stored samples from frontal cortex at this age. Surprisingly we observed an increase in GSH in the LPS group, which was dependent on sex, in the frontal cortex, but not in the hippocampus at PND 60. The significance of these findings is unclear at moment, but the increase in GSH could indicate a compensatory mechanism to the oxidative stress that occurs in this model.

Some limitations of this study should be noted. Firstly, MIA is a risk factor, not a model, as we have mentioned throughout the text, for several developmental neuropsychiatric disorders, including SCZ, autism and bipolar disorder. Secondly, we have focused this study on astroglial cells; however, other glial cells such as oligodendrocytes and microglia should also be investigated as these also contribute to the behavioral phenotypes of MIA observed at PND 60 (DF de Souza, unpublished results). Finally, astrocytes, as mentioned, are a heterogeneous group of cells, and the differences in levels of S100B and GFAP observed in the different cerebral regions may reflect this characteristic. Furthermore, numerous other specific astroglial parameters such as glutamate transporters, glutamine synthesis and GSH synthesis should be investigated in future studies to amplify the understanding of astroglial activity in MIA and SCZ pathology for diagnosis and even therapeutic intervention.

In summary, our results show that prenatal LPS challenge leads to neurochemical abnormalities in astroglial markers during postnatal life and these findings reinforce the hypothesis that MIA may underlie SCZ pathology. The S100B protein exhibited an age-dependent pattern of expression, being increased in the frontal cortex and hippocampus of the MIA group at PND 60, while at PND 30, male rats presented an increase in S100B levels only in the frontal cortex. Considering that S100B secretion is reduced by elevation of glutamate levels, we may hypothesize that this early increment in frontal cortex tissue of males is associated with elevated extracellular levels of glutamate and glutamatergic hypofunction, an alteration commonly associated with SCZ pathology. Accordingly, we also found augmented GFAP expression in the frontal cortex of the LPS group at PND 30, but not in the hippocampus. Moreover, we found a decrease in CSF levels of S100B in females at PND 30 in the LPS group, but not later on at PND 60. Taken together, data indicate that astroglial changes induced by MIA are dependent on sex and brain region, and that such changes could reflect astroglial dysfunction. Such dysfunction could help us, in part, to understand the abnormal neuronal connectivity and developmental aspects of SCZ and other psychiatric disorders.

## Author Contributions

DS and C-AG: Conception and experimental design, acquisition and analysis of data, and writing the manuscript. KW, PL, GB, LT, CB, RB: Experimental design, acquisition and analysis of data, and writing the manuscript.

## Conflict of Interest Statement

The authors declare that the research was conducted in the absence of any commercial or financial relationships that could be construed as a potential conflict of interest.

## Abbreviations

CNS: central nervous system
ELISA: enzyme-linked immunosorbent assay
GFAP: glial fibrillary acidic protein
GSH: glutathione
IL-6: interleukin-6
LPS: lipopolysaccharide
MIA: maternal immune activation
NeuN: neuronal nuclei
PND: postnatal day
ROS: reactive oxygen species
